# Supplementary material for: Complexome profiling of the Chlamydomonas psb28 mutant reveals TEF5 as an early PSII assembly factor
Source: Plant Cell. 2025 Mar 17;37(6):koaf055. doi: 10.1093/plcell/koaf055 (PMC12164590; doi:10.1093/plcell/koaf055)
Supplement: koaf055_Supplementary_Data [file koaf055_supplementary_data.zip › psb28_interactive.zip!/plots/intensity/qualityPlots/peptides/CCS1_Cre13.g575000.t1.1.html]

### Cre13.g575000.t1.1

Trivial name: CCS1  
SeqCoverage [median]: 26.60  
Mass: 64.90 kDa  
  
 

| Sequence | MissedCleavage | Mass [Da] | wtIntensity | muIntensity | Score |
| --- | --- | --- | --- | --- | --- |
| APRPTDAPSAIDDAPSTSGLGLGGGVASPR | 1 | 2789.39 | 0.00 | 4478.05 | 81.20 |
| DPQSVVFYDAK | 0 | 1267.61 | 0.00 | 2109.64 | 100.88 |
| FLTQPK | 0 | 732.42 | 0.00 | 2384.28 | 98.03 |
| GISILAR | 0 | 728.45 | 0.00 | 376.07 | 82.26 |
| GQFVGVR | 0 | 761.42 | 456.15 | 1121.04 | 120.90 |
| GYQVFVK | 0 | 839.45 | 0.00 | 937.04 | 72.02 |
| LGNTPPTLDVLK | 0 | 1266.72 | 451.98 | 2469.80 | 81.02 |
| TAFNLPMASLEGKPGVAGR | 1 | 1915.00 | 0.00 | 3259.39 | 105.38 |
| TEVLPNAR | 0 | 898.49 | 1543.74 | 776.77 | 120.52 |
| VLGQDAPLAR | 0 | 1038.58 | 0.00 | 3253.43 | 67.90 |
|  |  |  | 2451.9 | 21165.5 |  |
